# West Nile Virus Lineage 2 from Blood Donor, Greece

**DOI:** 10.3201/eid1804.110771

**Published:** 2012-04

**Authors:** Anna Papa, Constantina Politis, Athina Tsoukala, Aikaterini Eglezou, Vassiliki Bakaloudi, Maria Hatzitaki, Katerina Tsergouli

**Affiliations:** Aristotle University of Thessaloniki, Thessaloniki, Greece (A. Papa, K. Tsergouli);; National Coordinating Haemovigilance Centre of the Hellenic Centre for Disease Control and Prevention, Athens, Greece (C. Politis);; AHEPA University General Hospital Blood Centre, Thessaloniki (A. Tsoukala, V. Bakaloudi);; Papanikolaou General Hospital Blood Bank, Thessaloniki (A. Eglezou);; Koutlibaneio General Hospital Blood Centre, Larissa, Greece (M. Hatzitaki)

**Keywords:** West Nile virus, lineage 2, blood donor, viruses, vector-borne infections, Greece

**To the Editor:** West Nile virus (WNV) is a mosquito-borne flavivirus that primarily causes an asymptomatic or mild disease in humans; however, in <1% of infected persons, it causes neurologic disease. The virus has received increased attention since 2002 when it was established that WNV is transmissible by blood transfusion and organ transplantation (*1*).

A major WNV outbreak occurred in 2010 in Greece; most cases occurred in the northern part of the country (*2*). Of the 197 WNV neuroinvasive cases reported, 33 were fatal (*3*). Many nonneuroinvasive cases were observed (*4*). A lineage 2 WNV (Nea Santa-Greece-2010 strain) was detected in *Culex pipiens* mosquitoes collected at 2 locations where WNV cases had been reported (*5*). Although this strain shows high genetic identity to a Hungarian WNV strain isolated from birds in 2004, it has the amino acid substitution H_249_P in nonstructural protein 3 (NS3) (*6*). This mutation has been associated with increased virulence in WNV lineage 1 strains (*7*). Clinical WNV disease in humans had not been previously documented in Greece, and surveillance of blood donors in 2006 and 2007 did not show any WNV-positive result (*8*).

On August 11, 2010, shortly after confirmation of the outbreak of WNV infections in humans in Greece, an action plan for the protection of blood safety was initiated. All donors were asked to report any fever-like illness up to 15 days after donation. Individual donation nucleic acid testing (NAT) of all blood donors living in the WNV-affected areas was implemented on August 22, 2010.

The first WNV-positive by NAT result was obtained from a sample donated on August 22. Testing was performed by using the automated Procleix TIGRIS System (Chiron Corporation, Emeryville, CA, USA). The WNV-positive blood donor was a 40-year-old immunocompetent woman, a resident of a village in northern Greece. The village is located between 2 lakes, and the area is one of Europe’s major wetlands. The 2 locations where the WNV-positive mosquitoes were collected are near each other (Figure A1). The woman was working in an open-air fish market and reported numerous mosquito bites. At the time of blood donation, she was asymptomatic; 2 days later she had myalgia, arthralgia, and severe retro-orbital pain, lasting 2–3 days each. A second blood sample taken on August 26 was also NAT positive.

Serologic testing for WNV IgM and IgG was performed by ELISA (WNV IgM Capture DxSelect and WNV IgG DxSelect; Focus Diagnostics Inc., Cypress, CA, USA). An index ≥1.5 for IgM and ≥1.1 for IgG was considered positive. No antibodies were detected in the initial and second serum samples; however, a third sample taken on September 20 was positive for WNV IgM and IgG (indices 4.7 and 3.8, respectively).

Nested reverse transcription PCRs with the initial blood sample gave positive results (*9**,**10*). WNV lineage 2 sequences were obtained and were identical to those of the Nea Santa-Greece-2010 strain (*6*). One milliliter of each 1:10 and 1:100 dilution of whole blood in minimum essential medium containing antimicrobial drugs and 2% fetal bovine serum was placed on Vero E6 cell monolayers in 25-cm^2^ cell culture flasks. The procedure was performed in a biosafety level 3 laboratory, in which WNV lineage 2 had never been handled. After the sample was incubated 1 hour at 37°C in a 5% CO_2_ incubator, 9 mL of fresh medium containing 4% fetal bovine serum was added.

Cytopathic effects were observed in the flask inoculated with the 1:100 dilution on day 3 after infection. An aliquot of supernatant was used to infect fresh cell monolayers. WNV growth in the cell culture was demonstrated by reverse transcription PCR and immunofluorescence assay. Sequences of the cell culture isolate were identical to those of the directly detected virus.

To check whether the isolate possessed the H_249_P substitution, a set of nested primers spanning the region 5140–5660 from the WNV genome was designed: NS3a-5′-GCTGGCTTCGAACCTGA-3′ and NS3b-5′-CAATCATCGTTCTTGC-3′ for the first round PCR and NS3c-5′-GCTGCTGAGATGTCTGA-3′ and NS3d-5′-TCATATCCAGTGTTCCA-3′ for the nested PCR. The H_249_P substitution was present. Sequences were submitted to GenBank (accession nos. JF917091, JF917092).

Virus isolation from WNV patients is usually unsuccessful because viremia levels are low and last only a short time. WNV strains are usually isolated from immunocompromised patients, blood donors, IgM-negative immunocompetent patients who seroconverted, or autopsy brain samples. For the donor reported here, WNV was isolated 2 days before illness onset, when no antibodies were present. The WNV-positive blood donor was detected 1 day after the introduction of blood screening.

The early diagnosis of the initial human WNV cases in Greece, which resulted in prompt implementation of NAT testing, had a substantial positive impact on the safety of the blood supply in the affected areas. The risk for virus transmission was reduced for blood recipients, in particular those who receive multiple transfusions and immunocompromised patients in need of transfusion.

## Figures and Tables

**Figure A1:**
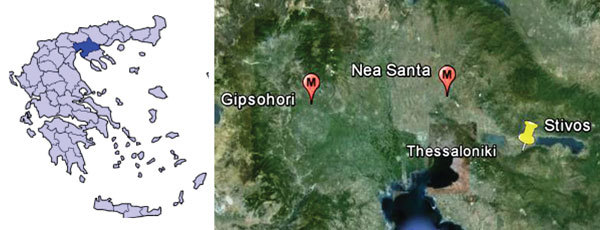
A) Region of Greece where West Nile virus (WNV) was found. B) Residence of the blood donor from whom WNV was isolated (yellow pin) and places where WNV-positive *Culex pipiens* mosquitoes were collected (red pins).
